# Advanced TiO_2_/Al_2_O_3_ Bilayer ALD Coatings for Improved Lithium-Rich Layered Oxide Electrodes

**DOI:** 10.1021/acsami.3c16948

**Published:** 2024-02-29

**Authors:** Wei-Ming Chen, Hsin-Yu Hsieh, Dong-Ze Wu, Horng-Yi Tang, Kuei-Shu Chang-Liao, Po-Wei Chi, Phillip M. Wu, Maw-Kuen Wu

**Affiliations:** † Institute of Physics, Academia Sinica, 128, Section 2, Academia Road, Taipei 11529, Taiwan; ‡ Nano Science and Technology Program, Taiwan International Graduate Program, Academia Sinica and National Tsing Hua University, 128, Section 2, Academia Road, Taipei 11529, Taiwan; § Department of Engineering and System Science, National Tsing Hua University, 101, Section 2, Kuang-Fu Road, Hsinchu 300044, Taiwan; ∥ Graduate Institute of Energy and Sustainability Technology, National Taiwan University of Science and Technology, 43 Keelung Road, Sec 4, Taipei 10607, Taiwan; ⊥ Department of Applied Chemistry, National Chi Nan University, 1 University Road, Puli, Nantou 545301, Taiwan; # College of Science, National Chung Hsing University, 145 Xingda Rd., South Dist., Taichung City 402, Taiwan

**Keywords:** lithium-ion battery, surface modification, lithium-rich cathode, atomic layer deposition, thin film

## Abstract

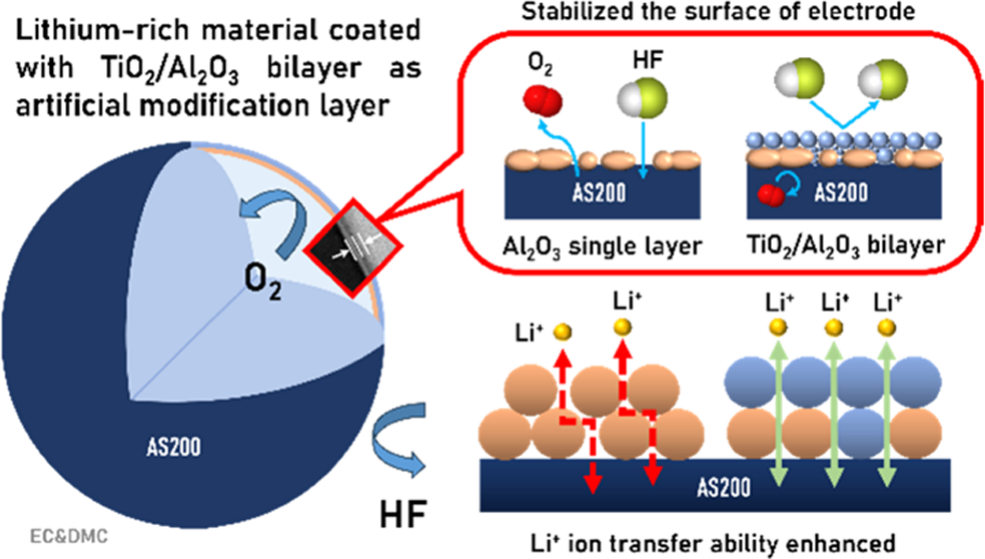

Surface modification is a highly effective strategy for addressing issues in lithium-rich layered oxide (LLO) cathodes, including phase transformation, particle cracking, oxygen gas release, and transition-metal ion dissolution. Existing single-/double-layer coating strategies face drawbacks such as poor component contact and complexity. Herein, we present the results of a low-temperature atomic layer deposition (ALD) process for creating a TiO_2_/Al_2_O_3_ bilayer on composite cathodes made of AS200 (Li_1.08_Ni_0.34_Co_0.08_Mn_0.5_O_2_). Electrochemical analysis demonstrates that TiO_2_/Al_2_O_3_-coated LLO electrodes exhibit improved discharge capacities and enhanced capacity retention compared with uncoated samples. The TAA-5/AS200 bilayer-coated electrode, in particular, demonstrates exceptional capacity retention (∼90.4%) and a specific discharge capacity of 146 mAh g^–1^ after 100 cycles at 1C within the voltage range of 2.2 to 4.6 V. The coated electrodes also show reduced voltage decay, lower surface film resistance, and improved interfacial charge transfer resistances, contributing to enhanced stability. The ALD-deposited TiO_2_/Al_2_O_3_ bilayer coatings exhibit promising potential for advancing the electrochemical performance of lithium-rich layered oxide cathodes in lithium-ion batteries.

## 1 Introduction

In recent years, there has been a notable increase in the adoption of electric vehicles (EVs) and hybrid electric vehicles (HEVs) so that rechargeable lithium-ion batteries (LIBs) have become widely utilized in public transportation.^1^ With the increasing demands in the transportation sector, studies on rechargeable lithium-ion batteries (LIBs) with the aim of achieving extended longevity and enhanced energy storage capacity have become an important research topic. Fundamentally, the energy storage capacity of LIBs hinges on the difference in lithium chemical potential and the reversible capacity between the anode and cathode. However, in comparison to the anode, contemporary cathodes employed in mass-produced LIBs typically exhibit lower capacity. Over the past few decades, various cathode materials, including LiCoO_2_ and LiMnO_2_,^2^ layer-structured LiNi_1–*x*_Co_*x*_O_2_,^3^ spinel-structured LiMn_2_O_4_^4^ and LiNi_0.5_Mn_1.5_O_4_,^5^ and olivine-structured LiFePO_4_^6^ have been developed for practical applications. However, the overall energy storage capacity of these batteries often faces limitations.

Achieving greater energy storage capacity and the ability to deliver more power in LIBs is crucial, necessitating the use of cathode materials with higher specific capacities. Among all the materials used for cathodes, lithium-rich layered oxides (LLO) hold significant potential,^7^ which could provide the high energy density required. Thus, LLO has been comprehensively applied in the commercialization of LIBs due to its superb capacity and ease of production. Nevertheless, LLO cathodes still face challenges. One significant issue is the presence of a long potential plateau at around 4.5 V during delithiation of the Li_2_MnO_3_ domains, accompanied by the release of oxygen from the lattice. This released oxygen leads to structural defects and phase transformation.^8^ To address this challenge, Ku et al.^9^ introduced a surface modification approach. They achieved the goal by applying a precoating of dopamine to construct a layered-spinel heterostructured Li-rich cathode material, followed by a heat treatment in air. This innovative technique yielded improvements in both the microstructure and electrochemical performance. Another noteworthy advancement came from Zhang et al.,^10^ who established heterojunction interfaces by employing the sol–gel method. Their work focused on the creation of bonds between Li_1.2_Ni_0.2_Mn_0.6_O_2_ and LaFeO_3_. The resulting Li_1.2_Ni_0.2_Mn_0.6_O_2_, featuring chemical bonding with perovskite LaFeO_3_, exhibited a remarkable capacity of 189.5 mAh g^–1^, with an outstanding 96.6% capacity retention after 150 cycles at 1C. Additionally, it achieved a discharge energy density surpassing 550 mWh g^–1^. This enhanced cycling stability can be attributed to the moderation of anion redox on the Li-rich surface and the suppression of surface oxygen release due to chemical bonding between LaFeO_3_ and Li_1.2_Ni_0.2_Mn_0.6_O_2_. Furthermore, Li et al.^11^ significantly improved the structural stability of Li_1.2_Ni_0.13_Co_0.13_Mn_0.54_O_2_ by introducing a lattice coherent epitaxial spinel stabilizer and implementing appropriate sulfur doping. This epitaxial spinel stabilizer, combined with beneficial oxygen vacancies, effectively maintained activated oxygen at the cathode surface, resulting in an enhanced structural stability even after extended cycling periods.

Furthermore, released oxygen is also highly reactive and can interact with the electrolyte, forming a product at the cathode-electrolyte interface (CEI). The CEIs formed with accustomed liquid electrolytes tend to be heterogeneous and susceptible to cracking during cycling. Consequently, the rate capability, thermal stability, and initial Coulombic efficiency of the battery are negatively affected.^12^ Moreover, the decomposition of LiPF_6_ salt from electrolyte at high potentials (>4 V vs Li/Li^+^) leads to large amounts of HF which attacks LLO to dissolve transition metals. Thus, LLO cannot offer an excellent cycle stability during cycling.

Therefore, the application of cathode surface coatings emerges as a crucial and effective strategy for enhancing various aspects of battery performance, including initial Coulombic efficiency, rate capability, capacity retention, and voltage stability during extended cycling. The primary function of these coatings is to act as an interphase that prevents direct chemical or redox reactions between the cathode material and the components in the electrolyte solution. In addition, they serve to block the release of molecular oxygen and the dissolution of transition metal ions.^13,14^ However, it is important to note that these coatings must also facilitate the rapid bidirectional transport of lithium ions between the cathode material and the electrolyte solution. To fulfill this role, they need to exhibit structural, thermal, and chemical stability over a wide range of potentials (typically 2–5 V vs Li) and enable high mobility of lithium ions.^15^

Thus, metal oxides stand out as promising candidates for cathode surface coatings, as they shield cathode materials from unwanted side reactions preventing oxygen release from the LLO and simultaneously enable the rapid transport of lithium ions.^16^ Numerous reports have explored the use of metal oxide materials as coating layers in battery technology. For example, Kobayashi et al.^17^ demonstrated that Al_2_O_3_ coating and precycling improved the high-temperature cycling performance of LLO cathodes. Mu et al.^18^ utilized WO_3_ coatings prepared with an ammonia solution, resulting in an increase in the first cycle Coulombic efficiency from 72 to 92.5% and offering protection against HF. Song et al.^19^ employed the sol–gel method and wet chemical process to successfully coat Li_1.2_Mn_0.54_Ni_0.13_Co_0.13_O_2_ materials with TiO_2_. This led to an initial Coulombic efficiency increase from 75.9 to 80.8% at 0.1C, with an initial discharge-specific capacity of 276.5 mAh g^–1^. After 200 cycles at 2C, the capacity retention rate of coated TiO_2_ reached 87.8%. Most of the reported cathode coating methods are based on liquid-phase deposition, though it is convenient and effective, this approach has its drawbacks. The deposition rate can be challenging to control and may result in uneven surface layer deposition. Additionally, the crystallization level of the coating layer may be suboptimal, necessitating further sintering treatment at elevated temperatures. This, in turn, increases energy consumption and processing costs.^20^ Despite significant improvements in these reported works, these methods often involve relatively complicated steps, particularly in the case of bilayer coating.^21−23^ To address the challenges previously mentioned, Atomic Layer Deposition (ALD) presents a promising solution. ALD is a precise method for applying ultrathin surface films to materials and particles, controlling the coating thickness at the atomic level. For instance, Yan et al.^24^ utilized ALD to coat Li_1.2_Ni_0.13_Co_0.13_ Mn_0.54_O_2_ with Al_2_O_3_. Their analysis, including electron microscopy and energy-dispersive spectrometry (EDS), demonstrated the presence of a consistently thin and uniform Al_2_O_3_ layer on the particle surface, even after numerous cycles. In contrast, the uncoated electrodes after cycling developed surface films consisting of carbonaceous and phosphorus-based species. In another study, Gao et al.^25^ employed CeO_2_ ALD coating to modify the surface of Li_1.2_Ni_0.13_Co_0.13_Mn_0.54_O_2_. This modification not only improved the substrate conductivity but also acted as a barrier to prevent metal dissolution. Under conditions of 1C and 55 °C, within a voltage range of 2.0–4.8 V, they achieved an initial capacity of 199 mAh g^–1^, representing an 8% increase over uncoated LLO particles. Even after 400 charge–discharge cycles, 60.2% of the initial capacity was retained, in stark contrast to that of the uncoated sample, which retained only 22% of its capacity after just 180 cycles of charge–discharge. While single-layer ALD coatings on LLO cathodes have received significant attention, research work on bilayer or multilayer configurations is lacking. Exploring these multilayer approaches could lead to significant advancements in battery technology.

From our previous work, we focus on a lithium-rich material, Li_1.083_Ni_0.333_Co_0.083_Mn_0.5_O_2_ (AS200), which has a reduced lithium content to modify the initial band structure. This modification results in around 80% of the capacity contributed by cationic redox reactions and 20% by anionic redox reactions.^26^ Additionally, we observed that the cation oxidation process in AS200 is slower in comparison to that in the typical Li-rich layered oxide (Li_1.2_Ni_0.13_Co_0.13_Mn_0.54_O_2_). This difference in kinetics and cation oxidation potential is likely due to local coordination variations related to different Li/O ratios.^27^ These findings offer valuable insights into how local electronic structure influences reaction mechanisms and kinetics, enhancing our understanding and control of LLO cathodes. However, this AS200 material exhibits a weakness in terms of its poor rate performance. We believe that this limitation is associated with the formation of a passivation layer on the AS200 surface during charge–discharge cycles. Therefore, it would be intriguing to explore the potential of using ALD modification with AS200 as a novel cathode design for LIBs. Following the works by Zhang et al.,^28^ they found that the TiO_2_ layer exhibited increased reactivity with lithium, forming a Li_*x*_TiO_2_ interface, thereby generating Ti^3+^ surface cations. These cations were identified as potential catalysts for reactions exacerbating capacity fading. Thus, we present the results of low-temperature ALD, which involved applying a TiO_2_/Al_2_O_3_ bilayer coating to the AS200 cathode. The TiO_2_/Al_2_O_3_-coated AS200, with a coating thickness of 5 nm, exhibited significantly improved capacity retention and high-rate performance compared to uncoated AS200, even under moderate cycling conditions. We have characterized the morphology and chemical structure of the TiO_2_/Al_2_O_3_ bilayered films using high-resolution transmission electron microscopy (HR-TEM) and X-ray photoemission spectroscopy (XPS).

## 2 Experimental section

### 2.1 AS200 Electrode Preparation

The cathode material, Li_1.08_Ni_0.34_Co_0.08_Mn_0.5_O_2_, was synthesized through a spray pyrolysis process using a 1000 mL aqueous solution containing transition metal nitrates (0.34 M Ni(NO_3_)_2_, 0.5 M Mn(NO_3_)_2_, 0.08 M Co(NO_3_)_2_) as the starting material. The resulting dry powder served as the precursor material. To produce Li_1.08_[Ni_0.34_Co_0.08_Mn_0.5_]O_2_, the precursor was subjected to ball milling along with Li_2_CO_3_, maintaining a molar ratio of Li:(Ni+Co+Mn) at 1.08:1.00. Subsequently, the mixture was heated under a flow of oxygen gas at 910 °C for 10 h, with a gradual ramp-up of temperature at a rate of 5 °C/min. This process ultimately yielded a spherical-shaped stoichiometric cathode material, referred to as AS200.

### 2.2 TiO_2_/Al_2_O_3_ Bilayered ALD Coating

TiO_2_/Al_2_O_3_ bilayered films were deposited onto AS200 electrodes by using the BENEQ TFS 500 atomic layer deposition (ALD) system. To begin, AS200 electrodes were prepared by blending the AS200 with conductive carbon (KS6 and Super P) and poly(vinylidene fluoride) (PVDF) in weight ratios of 91:2:2:5. Following this, *N*-Methyl-2-pyrrolidone (NMP) was introduced to create a uniform, black, and viscous slurry. This slurry was evenly coated onto aluminum foil using a doctor blade and subsequently dried under vacuum conditions at 110 °C for a duration of 24 h. Next, the AS200 electrode was placed into the ALD reactor, operated under a base pressure of around 1 mTorr. Here, 1.5 nm (and/or 2.5 nm)-thick Al_2_O_3_ thin films were first deposited onto the AS200 electrode using ALD cycles with trimethyl aluminum (TMA) and H_2_O as precursors at a temperature of 110 °C. Subsequently, 1.5 nm (and/or 2.5 nm)-thick TiO_2_ layers were deposited on top of the Al_2_O_3_ layer using ALD cycles with titanium tetrachloride (TiCl_4_) and H_2_O as precursors, still maintaining the deposition temperature at 110 °C. Precise control was achieved by setting the precursor dose and purge time for both Al_2_O_3_ and TiO_2_ ALD processes at 0.3 and 0.4 s, respectively, with 2 s N_2_ purge applied between each pulse. Furthermore, the growth rate for each Al_2_O_3_ and TiO_2_ layer was maintained at 1 and 1.5 Å/cycle, respectively. This meticulous deposition process resulted in the creation of 3 and 5 nm thick TiO_2_/Al_2_O_3_ bilayered films on the AS200 electrode, denoted as TAA-3 and TAA-5.

### 2.3 Lithium-Ion Battery Assembly and Measuring

CR2032 coin cells were carefully assembled in an argon-filled glovebox (Vigor). All materials used in the assembly process were predried at 110 °C in a vacuum oven. For the half-cells, the following components were utilized: an Ø13 mm cathode electrode, a lithium–metal anode with a Ø15 mm diameter, and a polypropylene separator sourced from Celgard Co. in Japan. The separator was soaked with a standard electrolyte solution consisting of 200 μL of a 1.0 M LiPF_6_ solution in a 1:1 by weight mixture of ethylene carbonate (EC) and dimethyl carbonate (DMC) obtained from Formosa Co. in Taiwan. To evaluate the electrochemical properties, CR2032-type coin cells were employed with metallic lithium serving as both the counter and reference electrode. Cyclic voltammetry tests were conducted at a scan rate of 0.1 mV s^–1^ at room temperature using a PARSTAT MC 200 electrochemistry workstation, with the measurement range set between 2.2 and 4.6 V. Charge and discharge profiles were obtained through galvanostatic cycling between 2.2 and 4.6 V vs Li^+^/Li, applying a constant current at a rate of 0.1C at room temperature via a Think Power battery testing system. Electrochemical impedance spectroscopy (EIS) was carried out using the same workstation for the CR2032-type coin cells, applying an AC amplitude of 10 mV. The measurement encompassed a frequency range spanning from 100 kHz to 0.1 Hz. The distribution of relaxation times (DRT) was calculated using DRTtools,^29,30^ based on the Gaussian discretization method with a regularization parameter of 10^–4^ and a full width at half-maximum (FWHM) of 0.5. Additionally, the Impedance Spectroscopy Genetic Programming (ISGP) program^31^ was utilized for further analysis.

### 2.4 Material Characterization

The crystal structure analysis of all prepared samples was conducted using powder X-ray diffraction (XRD) with Cu Kα (λ = 1.5406 Å) radiation in the 2θ range from 10 to 80°. This characterization was carried out by using a Rigaku XRD instrument from Japan. Furthermore, the morphology of all of the samples was assessed using a field emission scanning electron microscope (FE-SEM, FEI Inspect-F SEM). To determine the content of transition-metal species within all samples after cycling, X-ray photoelectron spectroscopy (XPS) was employed. This analysis utilized Al Kα radiation and was performed by using the ULVAC-PHI (Quantes) instrument. Data were collected and averaged from ten individual cells. Microstructure analysis was conducted by using a high-resolution transmission electron microscope (TEM, JEOL JEM-2100F). To safeguard the integrity of the TiO_2_/Al_2_O_3_ coating during sample preparation and subsequent processing, a protective layer of platinum was applied. This protective layer was created through an initial electron-beam deposition followed by ion-beam-assisted deposition, employing an SEIKO SMI3050SE Dual-Beam Focused Ion Beam (FIB) instrument. Following the application of a protective layer, TEM specimens were carefully prepared using gallium-focused ion-beam milling techniques.

## 3 Results and discussion

**Figure 1:**
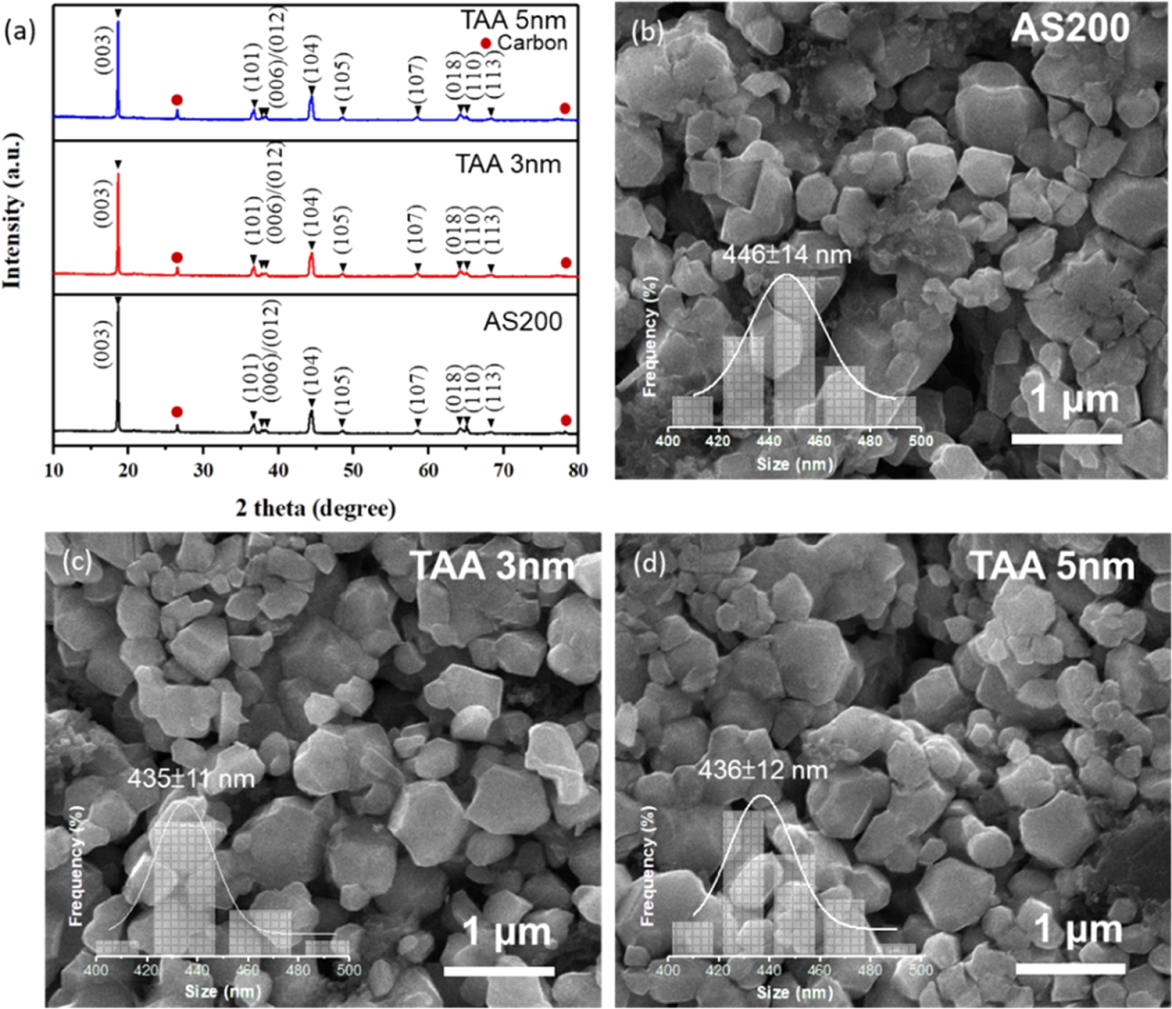
(a) XRD patterns of the AS200, TAA-3, and TAA-5 electrodes. Top-view SEM images for (b) AS200, (c) TAA-3, and (d) TAA-5 electrodes. Insets in panels (b) to (d) represent the corresponding particle size distributions.

In Figure 1a, X-ray diffraction (XRD) patterns of AS200, TAA-3, and TAA-5 samples are presented to understand structural changes following the application of ALD TiO_2_ and Al_2_O_3_ bilayer coatings on LLO. Before delving into the impact of these bilayer coatings, refer to Figure S1 in the Supporting Information for XRD patterns of single-layer samples (TiO_2_/AS200 and Al_2_O_3_/AS200). The XRD patterns reveal that all samples, including the single-layer references and bilayer-coated samples, exhibit similar diffraction peaks. This similarity suggests that the application of ALD TiO_2_ and Al_2_O_3_ bilayer coatings did not induce significant structural changes. The dominant peaks align well with the *R*3̅*m* crystal structure.^32^ Weak diffraction peaks observed at 20–30° are attributed to a lattice superstructure resulting from the arrangement of Mn ions in Li–Mn layers, a common feature in Li-rich materials belonging to the *C*2/*m* space group.^33^ Additionally, the resolved (006)/(102) and (018)/(110) peaks signify an ordered arrangement of transition metal ions within the layered oxides.^34,35^ The intense (003) peak, compared to (104), indicates the well-aligned nature of these layered oxides. Notably, distinct diffraction peaks corresponding to TiO_2_ and Al_2_O_3_ are absent, suggesting their thin and amorphous nature as coating layers.

**Figure 2:**
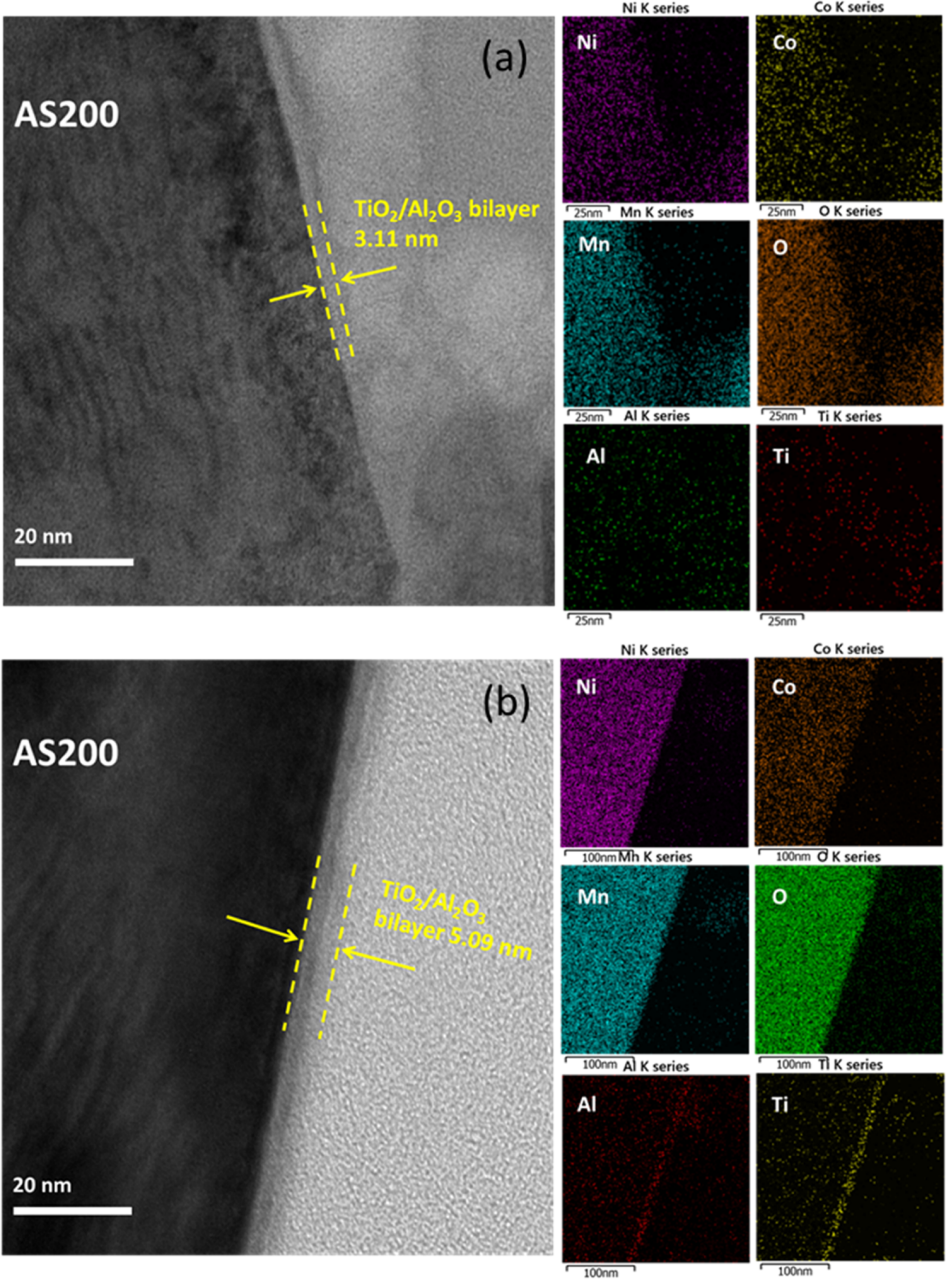
TEM images of the (a) TAA-3/AS200 and (b) TAA-5/AS200 electrodes. Elemental mapping images corresponding to (a) and (b) are displayed on the right side.

Figure 1b–d presents the SEM images of the AS200, TAA-3, and TAA-5 samples. These images reveal the presence of polygonal-shaped layered oxides, with dimensions falling within the range of 420 to 460 nm. However, the precise connection between the ultrathin layer and LLO remains somewhat elusive, primarily because of the significant disparity in their respective dimensions. To probe the surface morphology and elemental composition of these ultrathin layers, HR-TEM and EDS mappings are employed, as shown in Figure 2. First, the Al_2_O_3_ and TiO_2_ single-layer-coated LLO were examined (Figure S2a,b). Upon examination, it can be observed a well-defined boundary and the absence of lattice fringes on the surfaces of both samples. Significantly, the thickness measured at 5 nm corresponded precisely to the growth rate of TiO_2_ ALD, which is 1 Å/cycle. In contrast, for the Al_2_O_3_-coated samples, aggregated particles were present, and their thickness was also measured around 5 nm, aligning with the growth rate of the Al_2_O_3_ ALD, quantified at 1.5 Å/cycle. The underlying reasons for these distinct observations can be attributed to differences in the nucleation and growth modes during the ALD processes for TiO_2_ and Al_2_O_3_. During the ALD of TiO_2_, the initial stages entail the formation of a complete amorphous film during nucleation, followed by subsequent particle aggregation to facilitate film growth. Conversely, the ALD process for Al_2_O_3_ predominantly features island growth, driven by nonideal surfaces that create irregular nucleation sites for Al_2_O_3_.^36^ As the deposition proceeds, the presence of additional hydrogen-bonded H_2_O on the hydroxylated Al_2_O_3_ leads to an accelerated growth rate and the development of a discontinuous layer on the surface. Therefore, the preferred structure design involves the utilization of island-like Al_2_O_3_ as the bottom layer, with subsequent deposition of TiO_2_ on top. This arrangement facilitates gradual merging of the two layers, resulting in a smooth surface, as shown in Figure 2a,b. The composition analysis of the Al_2_O_3_ and TiO_2_ single-layer-coated LLO was conducted using TEM-energy dispersive X-ray spectroscopy (EDX) image mapping, as shown in Figure S2c,d. In both samples, identical signals corresponding to Mn, Ni, Co, and O, characteristic of lithium-rich layered oxides, were detected uniformly across the entire surface of the particles. Furthermore, in the corresponding high-angle annular dark field scanning transmission electron microscopy (HAADF-STEM) images, shown in Figure S2c,d, it is evident that the image intensity (contrast) in HAADF mode is directly proportional to the atomic number (*Z*). As a result, Al_2_O_3_ and TiO_2_ coatings exhibited a very low contrast in this mode. Significantly, both Al and Ti signals were identified and found to be uniformly distributed on the surfaces of the Al_2_O_3_ and TiO_2_ single-layer-coated LLO. This consistent result was also observed in the TAA bilayer samples, as shown in Figure 2a,b.

**Figure 3:**
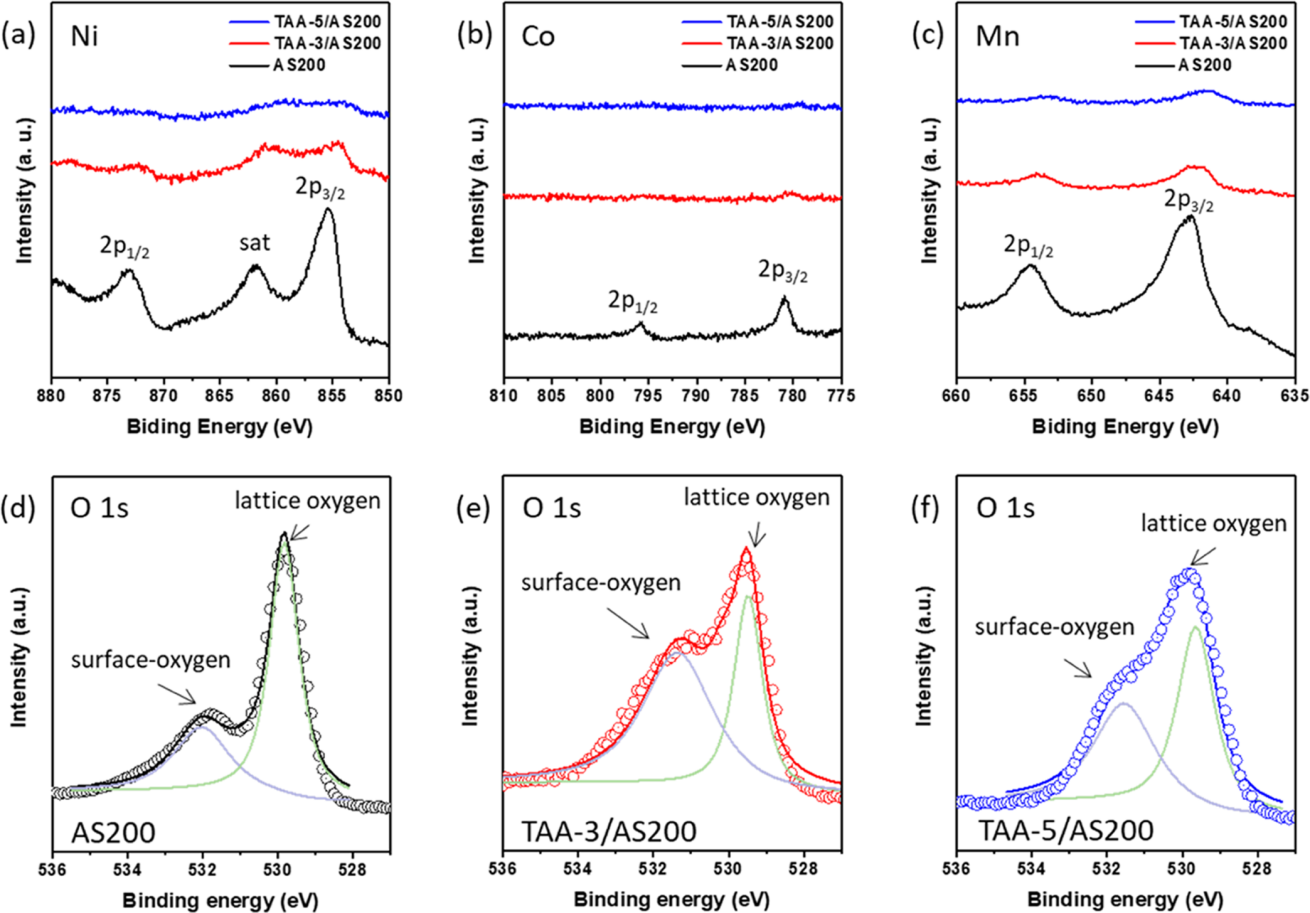
XPS spectra of (a) Ni 2p, (b) Co 2p, and (c) Mn 2p for AS200, TAA-3/AS200, and TAA-5/AS200 electrodes. (d–f) O 1s spectra for AS200, TAA-3/AS200, and TAA-5/AS200 electrodes, respectively.

After confirmation of the presence of Al and Ti elements on the sample surfaces, XPS was utilized to investigate the chemical composition of the species that had formed on the surfaces. In accordance with the norm, the XPS spectra of the three samples all displayed 2p_1/2_ and 2p_3/2_ core-level peaks corresponding to the Mn, Co, and Ni cations, as shown in Figure 3a–c. It is noted that after the coating of the TAA layer, the AS200 exhibited weaker peak intensities compared to the uncoated sample. The intensities decreased with an increasing thickness of the TAA layer. This reduction in intensity can be attributed to the lower content of the transition metals near the outermost surface of the coated samples. Furthermore, as shown in Figure 3a, XPS analysis of the Ni 2p_3/2_ spectra for these samples reveals a binding energy of 855.7 eV. This value closely matches that of Ni^3+^ in bulk LiNiO_2_, as opposed to Ni^2+^ which typically exhibits a biding energy of 854 eV.^37^ This observation is consistent with the findings in reported studies of similar layered compounds.^38,39^ One possible mechanism behind this phenomenon is the tendency of excess Li ions at the surface to occupy sites within the transition metal layers of the layered structure. This can lead to an increase in the oxidation state of Ni^2+^ as a compensatory measure to balance the overall charge. On the other hand, as shown in Figure 3b, the Co 2p_3/2_ binding energy is measured at 780.8 eV, a value matches to the reported binding energy of Co^3+^ in layered LiMO_2_ (where M stands for Ni, Co, and Mn).^40,41^ In Figure 3c, the Mn 2p spectra reveal a Mn 2p_3/2_ peak that fits well as a single peak with a binding energy of 642.7 eV. This observation is consistent with the presence of Mn^4+^ ions, as observed in manganese-based layered compounds.^42^ Additionally, an examination of the O 1s XPS spectra for AS200, TAA-3/AS200, and TAA-5/AS200 is presented in Figure 3d–f. A distinct peak at 529.4 eV corresponds to the lattice oxygen of LLOs.^43^ Furthermore, a peak at 531.6 eV indicates the presence of surface-oxidized species.^44^ Moreover, it is noted that the ALD coating layers, TAA-3 and TAA-5, form bonds with AS200 through these oxygen bridges.^45^

**Figure 4:**
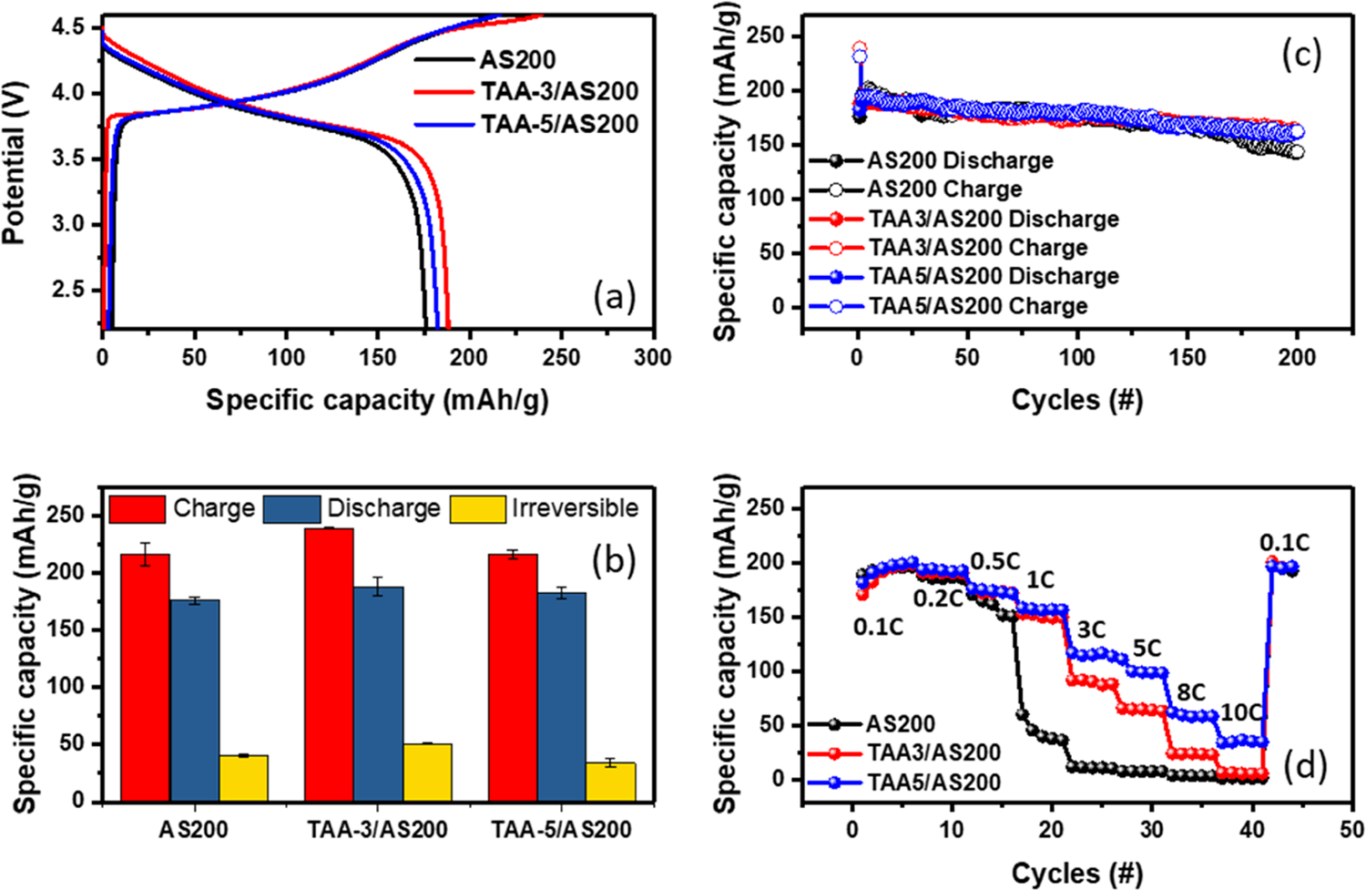
Electrochemical performance comparison of AS200, TAA-3/AS200, and TAA-5/AS200 electrodes. (a) Initial charge/discharge curves. (b) Corresponding histograms of the first charge, discharge, and irreversible capacity loss at 0.1C rate, with error bars representing one standard deviation calculated over three cells. (c) Cycle performance tested within the voltage range of 2.2 to 4.6 V at a rate of 0.1C. (d) Rate performances of AS200, TAA-3/AS200, and TAA-5/AS200 electrodes from 0.1 to 10C rate during 2.2 to 4.6 V.

Figure 4 displays the electrochemical performance of AS200, TAA-3/AS200, and TAA-5/AS200 electrodes tested within the voltage range of 2.2 to 4.6 V. Figure 4a presents the initial charge capacity of the AS200, TAA-3/AS200, and TAA-5/AS200 electrodes at 0.1C (216, 239, and 216 mAh g^–1^, respectively). The slightly reduced capacities in the TAA-5/AS200 electrodes can be attributed to the insulating nature of the coating. The initial charge/discharge curves for all three samples exhibit a slope region (3.9–4.36 V) associated with lithium-ion extraction and Ni^2+^ oxidation to Ni^+4^.^46^ It was known that when the voltage stays above 4.5 V for a long time, lithium ions are removed from AS200 to cause the formation of Li_2_MnO_3_. This will result in a significant loss of battery capacity and induces MnO_2_ impurity.^47^ The application of the TiO_2_/Al_2_O_3_ bilayer to AS200 results in an improved discharge capacity. The TAA-3/AS200 and TAA-5/AS200 electrodes demonstrate higher discharge specific capacities of 188 and 182 mAh g^–1^, respectively, compared to the pristine samples (176 mAh g^–1^), as shown in Figure 4b. Figure 4c shows the cycling performance of these samples under a current density of 0.1C over 200 charge–discharge cycles. Notably, all TiO_2_/Al_2_O_3_ bilayer-coated AS200 electrodes exhibit better specific discharge capacities and enhanced capacity retention compared with the pristine samples. Particularly noteworthy are the results for the TAA-3/AS200 samples, which demonstrate outstanding capacity retention (∼86%) and a specific discharge capacity of 162 mAh g^–1^, surpassing the performance of the pristine samples (∼72% and 143 mAh g^–1^, respectively).

Figures 4d and S3 demonstrate that the TAA series electrodes reveal markedly better C rate performance than AS200. At a rate of 0.5C, the performance of all samples is comparable. However, at 1C, the capacity of the TAA-3/AS200 and TAA-5/AS200 electrodes reaches 149 and 156 mAh g^–1^, respectively, significantly surpassing the 39 mAh g^–1^ measured for AS200 at 1C. This enhanced rate capability, observed in prior studies involving ALD-treated cathode materials such as Ni-rich-layered oxides (NMC)-based cathodes,^48−50^ LiMn_2_O_4_ cathodes^51^ and LiCoO_2_ cathodes,^52^ is believed to stem from the facile Li transport facilitated by the ALD-deposited layers. Reversible Li^+^ ion intercalation into LLO typically occurs through two-dimensional pathways.^53^ The ALD layer serves as a Li^+^ ion pathway, offering additional intercalation sites for Li^+^ ions in LLO, ultimately increasing battery capacity and supporting higher energy density.^54,55^ Furthermore, the rate performance for single-layer samples (TiO_2_/AS200 and Al_2_O_3_/AS200) have been verified and are shown in Figure S4 of the Supporting Information. Comparing them, TAA-coated samples exhibit better results, potentially attributed to fewer surface defects, such as pinholes, occurring on the electrode surface. Additionally, the cycling performance of TAA-coated samples under a current density of 1C with 100 charge–discharge cycles has been confirmed and is shown in Figure S5. Notably, the TAA-5 electrode demonstrated excellent capacity retention, reaching 90.4% after the cycling process.

**Figure 5:**
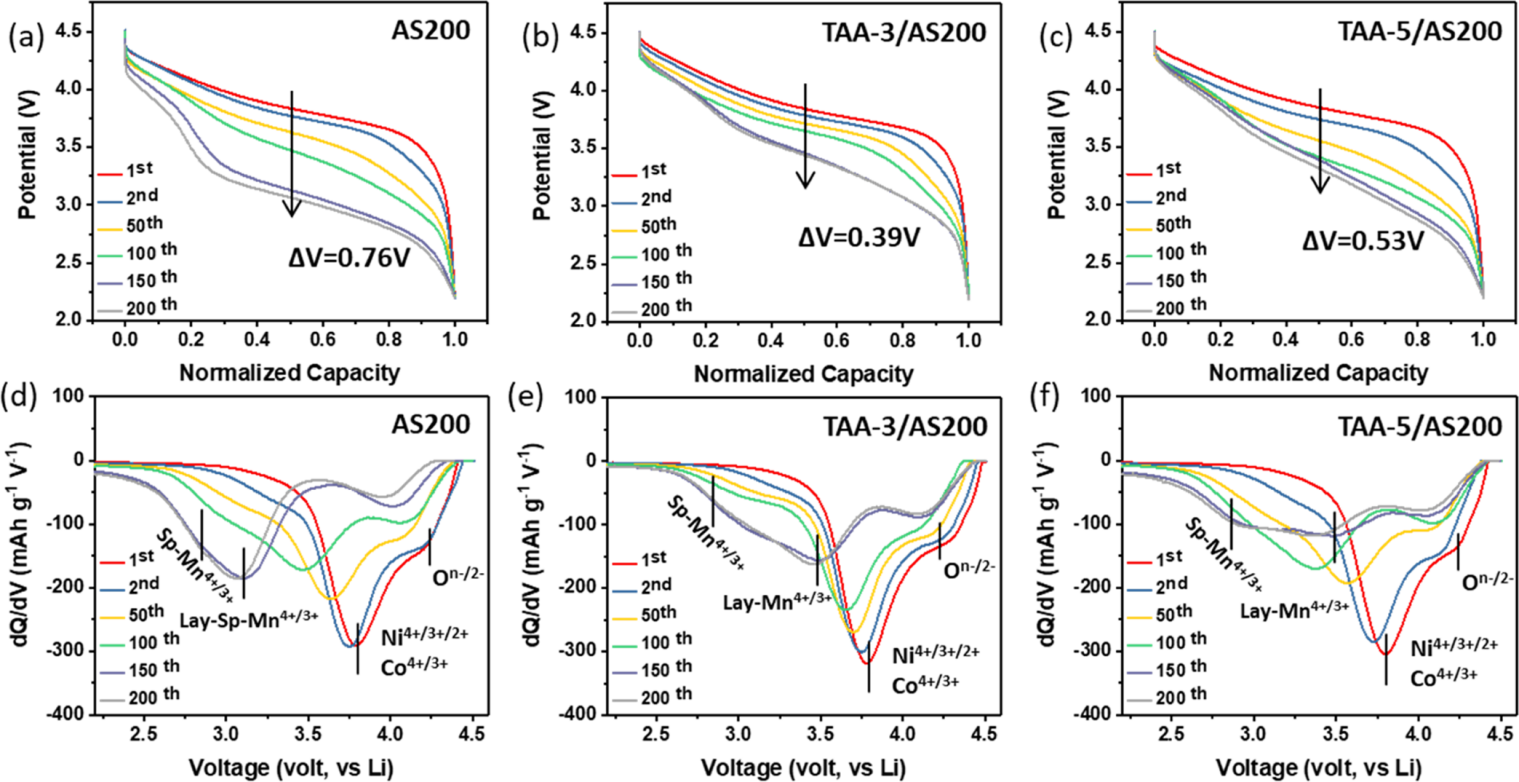
Voltage versus normalized capacity curves for the (a) AS200, (b) TAA-3/AS200, and (c) TAA-5/AS200 electrodes. Additionally, the differential capacity (d*Q*/d*V*) plots for these electrodes are shown in panels (d), (e), and (f) respectively.

Figure 5a–c display the voltage vs normalized capacity of the investigated electrodes at 0.1C rate over many cycles. The TAA series electrodes demonstrate better stability, which is evident in their resistance to voltage decay as observed in the evolution of their discharge profiles over 200 cycles. While all electrodes exhibited noticeable voltage decay in the long cycle, the AS200 electrode experienced increasingly severe decay with continued cycling. Specifically, the midpoint discharge voltage (MPV) of the AS200 electrode decreased by 760 mV, whereas the MPV of TAA-3/AS200 and TAA-5/AS200 electrodes decreased by only 390 and 530 mV, respectively. To better understand the observed voltage fade, we analyzed the d*Q*/d*V* plots of the samples with various cycles, as shown in Figure 5d–f. During the initial discharge cycle, a significant reduction peak is evident in all samples, occurring between 3.7 and 4.4 V. This peak corresponds to the reduction of Ni^4+^ to Ni^3+^/Ni^2+^, Co^4+^/Co^3+^, and O^*n*–^/O^2–^.^56^ As the charge–discharge cycles progress to 150 cycles, these reduction peaks only slightly weaken and shift to a lower potential. Surprisingly, in the AS200 electrode, all reduction peaks nearly vanish after 150 cycles and a strong peak at 3 V emerges. This peak is associated with the reduction of Mn from 4+ to 3+ in the layered oxide with spinel-like structures.^57^ In contrast, in the TAA-3/AS200 and TAA-5/AS200 electrodes, the shift in the reduction peak is less pronounced, halting at around 3.4 V. This corresponds to the reduction of Mn^4+^ to Mn^3+^ in layered structures.^57,58^ Additionally, a minor peak at around 2.8 V is observed in all samples, linked to the reduction of Mn^4+^ in the layered oxide with stronger spinel features. These results indicate that the transformation of the layered to spinel phases during cycling is more prohibited for the TAA series samples, resulting in less voltage fade and capacity loss. On the other hand, Figure S6 presents the SEM images of all samples after 100 cycles at 0.1C, revealing no significant differences in morphology. Concurrently, *ex-situ* XRD spectra indicate a reduced prominence of spinel phases in TAA-coated samples compared to that of the pristine sample, as shown in Figure S7. Notably, only the (003) diffraction peak exhibits weaker intensity in the sample without TAA coating, suggesting lithium deficiency and layered structure degradation in Li-rich cathodes, as proposed by Mohanty et al.^59^ Additionally, XPS spectra (Figure S8) of all TAA-coated samples after 100 cycles confirm the persistence of the Al 2p and Ti 2p peaks, indicating the enduring presence of the TAA coating layer. These findings affirm that the TAA coating inhibits structural degradation of the cathode material, remaining intact after 100 cycles of charge/discharge processes. The observed reasons and supporting evidence collectively affirm the enhanced structural stability exhibited by the TAA-coated samples throughout the cycling process.

**Figure 6:**
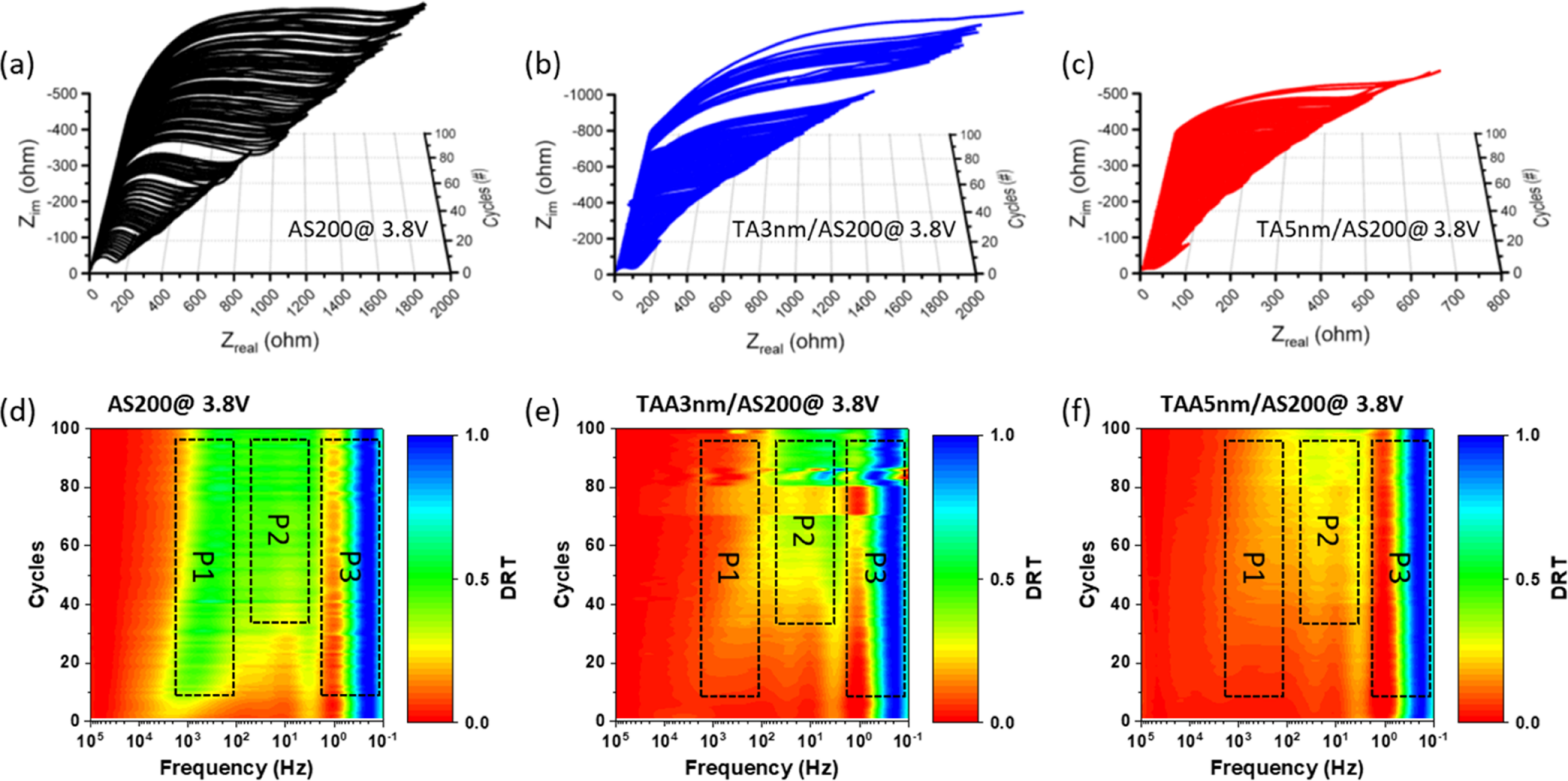
EIS spectra of (a) AS200, (b) TAA-3/AS200, and (c) TAA-5/AS200 recorded at 3.8 V with frequency ranging from 100 kHz to 0.1 Hz over 0 to 100 cycles. (d), (e), and (f) show the corresponding DRT color-mapped plots for AS200, TAA-3/AS200, and TAA-5/AS200 electrodes, respectively.

To gain more insight into the interfacial properties between the electrode and electrolyte, we carried out electrochemical impedance spectroscopy (EIS) measurements in the frequency range of 100 kHz to 0.1 Hz at MPV (3.8 V) after activation with 0.1C for 3 cycles. The results are shown in Figure 6a–c. All electrodes exhibit a compressed semicircle in the high-frequency region and an inclined line in the low-frequency region suggest that the electrochemical process is primarily influenced by charge transfer at the electrode/electrolyte interface and Li^+^ ion diffusion.^60^ Furthermore, a noticeable reduction in charge-transfer resistance was observed in the high-frequency semicircle of the TAA series electrode compared to that of the AS200 electrode. However, after 100 cycles, although the total impedance values of batteries with TAA series electrodes increased significantly, they remain much smaller than those with AS200 electrodes. To further understand the observed results, we conducted the distribution of relaxation time (DRT) analysis based on the impedance responses. The DRT derived from all the impedance data were mapped out in 2D color-mapped plots, as shown in Figure 6d–f. These plots revealed three distinct signatures around the frequency regions of 103 Hz (P1), 10 Hz (P2), and 1 Hz (P3) respectively. P1 can be attributed to the surface film resistance resulting from Li-ion migration through the electrode surfaces, P2 to the electrode/electrolyte interfacial charge transfer resistances, and P3 to the active material/current collector interfacial charge transfer and solid diffusion.^61−64^ Upon comparing the color-mapped plots of all samples, it is evident that the signatures P1 and P2 in the AS200 electrode are much brighter than in the TAA series electrode, indicating a higher resistance of surface film and interfacial charge transfer resistances in the AS200 electrode. The relatively minor variation in impedance observed in the TAA series electrode is due to the ability of the TiO_2_/Al_2_O_3_ bilayer coating to mitigate changes in the electrode/electrolyte interface in cycling. The slightly higher resistance of the coated material is associated with the smaller contact area between the active material and the electrolyte after the TiO_2_/Al_2_O_3_ bilayer coating.^65,66^ Consequently, the TAA series electrode displayed fewer structural changes, lighter grain coarsening, and smaller fluctuations in the impedance during cycling. These characteristics signify the efficient mitigation of discharge voltage fading through the TiO_2_/Al_2_O_3_ bilayer coating.

**Figure 7:**
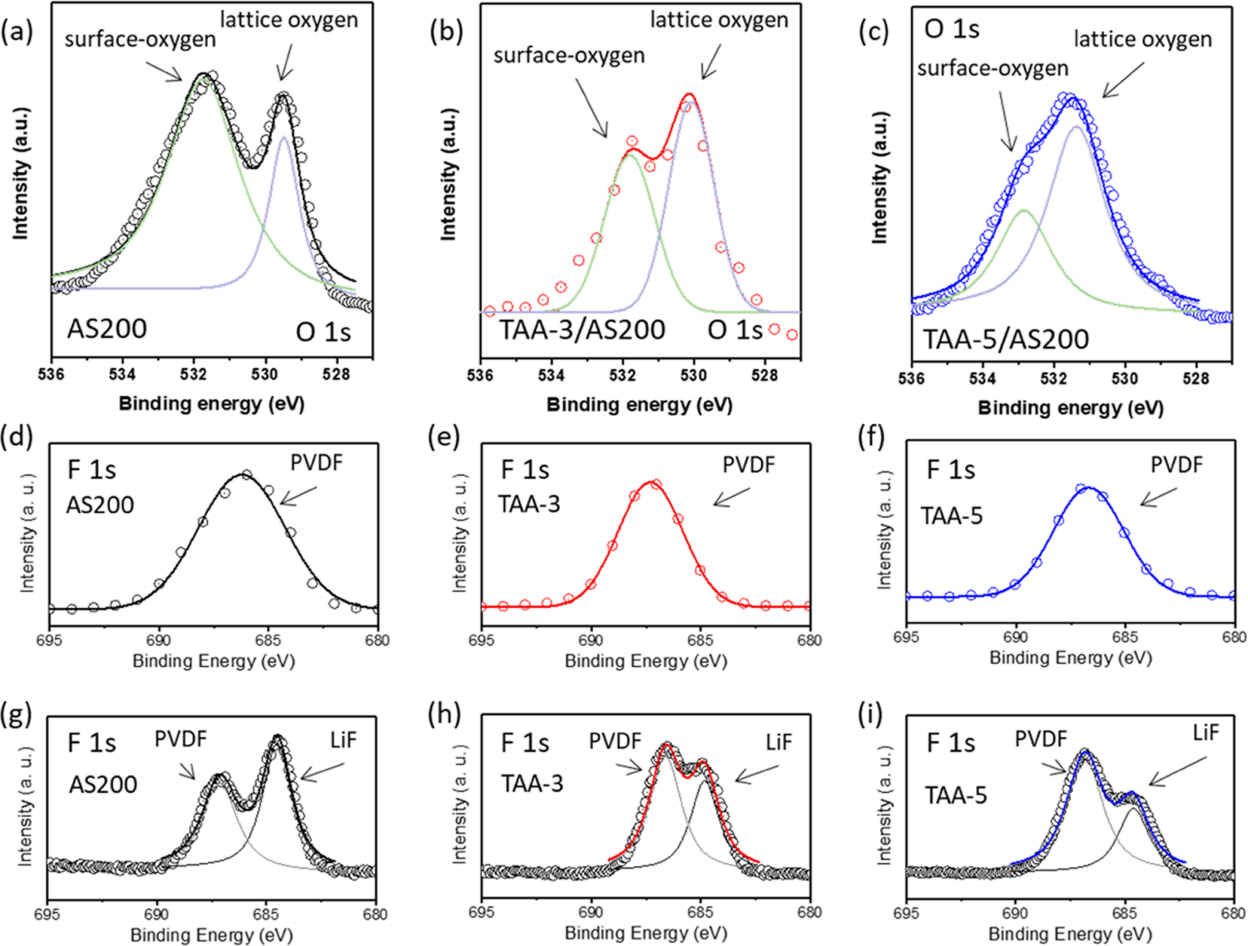
O 1s XPS spectra of (a) AS200, (b) TAA-3/AS200, and (c) TAA-5/AS200 electrode recorded after 100 cycles. (d–f) F 1s XPS spectra of AS200, TAA-3/AS200, and TAA-5/AS200 electrode at initial state. (g–i) F 1s XPS spectra of AS200, TAA-3/AS200, and TAA-5/AS200 electrode after 100 cycles.

The significant drop in surface film impedance observed in the results indicates less intense electrolyte decomposition in the TAA series electrode. This phenomenon is closely related to the protective shielding effect of the TiO_2_/Al_2_O_3_ bilayer, preventing from direct contact between AS200 active material and the electrolyte. Furthermore, the suppressed release of oxygen, a known facilitator of electrolyte decomposition and hydrolysis under high voltage conditions,^67^ could also contribute to this effect. To delve deeper into the surface compositions of the electrodes, XPS was measured. Figure 7a–c shows the O 1s spectra for AS200, TAA-3/AS200, and TAA-5/AS200 electrodes after 100 cycles at 0.1C, and the corresponding spectra before cycling are shown in Figure 3d–f. A close comparison between these figures reveals that after cycling, the peak at 531.6 eV, associated with the surface film, becomes more prominent indicating a thicker surface film due to increased electrolyte decomposition on the surface of the active material. In addition, the interaction between the electrolyte and the active material, forming metal fluoride byproducts, further contributes to the growth of the surface film. The XPS spectra of F 1s for AS200, TAA-3/AS200, and TAA-5/AS200 electrodes, before and after cycling, are shown in Figure 7d–i. Before cycling, all samples exhibited a characteristic peak at around 687 eV corresponding to C–F originating from the binder (PVDF). After 100 charge/discharge cycles at 0.1C, this peak weakened, and an additional peak at around 684 eV (black line) emerged, corresponding to F-anion resulting from MFx byproducts.^68^ It is noted that the surface of the coated sample showed less LiF, indicating that the presence of TiO_2_/Al_2_O_3_ bilayer coatings effectively protected the active material surface from reactions leading to LiF formation after exposure to HF.^69^

## 4 Conclusions

In summary, the comprehensive analysis of TiO_2_/Al_2_O_3_ bilayer-coated lithium-rich layered oxide (LLO) electrodes has provided valuable insights into their structural integrity and electrochemical performance. XRD patterns revealed that the TiO_2_/Al_2_O_3_ coatings did not induce significant structural changes in the LLO samples, affirming the stability of the crystal structure, even after the coating process. SEM images indicated well-defined polygonal-shaped LLO particles, with dimensions ranging from 420 to 460 nm. HR-TEM and EDS mapping confirmed the successful deposition of the thin TiO_2_/Al_2_O_3_ bilayer on the LLO surface. The TiO_2_/Al_2_O_3_ bilayer-coated LLO electrodes exhibited remarkable electrochemical performance improvements. Specifically, the TAA-3/AS200 samples demonstrated exceptional capacity retention, retaining approximately 86% of their initial capacity (162 mAh g^–1^) over 200 charge/discharge cycles, compared to the pristine samples that retained only about 72% of their initial capacity (143 mAh g^–1^). The initial charge capacity of TAA-3/AS200 electrodes at 0.1C was notably higher at 239 mAh g^–1^ compared to that of AS200 (216 mAh g^–1^), indicating enhanced energy storage capabilities. Furthermore, the rate capability of the TiO_2_/Al_2_O_3_-coated electrodes was significantly enhanced. At 1C, the TAA-3/AS200 and TAA-5/AS200 electrodes demonstrated impressive capacities of 149 and 156 mAh g^–1^, respectively, surpassing the 39 mAh g^–1^ measured for AS200 at the same rate. The TiO_2_/Al_2_O_3_ bilayer-coated electrodes displayed a 4-fold capacity improvement at 1C, highlighting the enhanced Li^+^ ion intercalation kinetics facilitated by the ALD-deposited layers. EIS results revealed reduced charge-transfer resistance in the high-frequency semicircle of the TiO_2_/Al_2_O_3_-coated electrodes compared with the uncoated AS200 electrode, indicating improved charge transfer at the electrode/electrolyte interface. The DRT analysis further supported the superior interfacial properties of the coated electrodes, with minimized changes in impedance during cycling. Moreover, XPS data indicated a thicker surface film due to increased electrolyte decomposition on the uncoated AS200 electrode compared with the TiO_2_/Al_2_O_3_-coated electrodes after cycling. The presence of TiO_2_/Al_2_O_3_ bilayer coatings effectively protected the active material surface from reactions, leading to LiF formation after exposure to HF, contributing to enhanced stability. In summary, the TiO_2_/Al_2_O_3_ bilayer coatings not only preserved the structural integrity of the LLO but also significantly enhanced its electrochemical performance. The coated electrodes exhibited superior capacity retention, enhanced rate capability, and improved stability, making them promising candidates for high-performance lithium-ion batteries. These findings underscore the effectiveness of ALD coatings in optimizing the surface properties of electrode materials, paving the way for the development of advanced energy storage devices.

## Supporting Information Available

XRD patterns/TEM images of AS200 with different single-layer coatings; cycling performance of all samples under 1C, along with corresponding charge–discharge curves at different cycles; XPS spectrum of all samples following 100 cycles at 0.1C within the voltage range of 2.2–4.6 V (PDF)
