# Supplementary material for: Advanced TiO2/Al2O3 Bilayer ALD Coatings for Improved Lithium-Rich Layered Oxide Electrodes
Source: ACS Appl Mater Interfaces. 2024 Feb 29;16(10):13029–40. doi: 10.1021/acsami.3c16948 (PMC10941074; doi:10.1021/acsami.3c16948)
Supplement: Supplementary file 1 — am3c16948_si_001.pdf [file am3c16948_si_001.pdf]

Supporting Information

Advanced TiO_2/Al_2O_3 Bilayer ALD Coatings for Improved Lithium-Rich Layered Oxide Electrodes

Wei-Ming Chen[a, b, c], Hsin-Yu Hsieh[a], Dong-Ze Wu[a, d], Horng-Yi Tang[e], Kuei-Shu Chang-Liao[c], Po-Wei Chi[a], Phillip M. Wu[a, f]* and Maw-Kuen Wu[a]*

[a] Institute of Physics, Academia Sinica

128, Section 2, Academia Road, Taipei 11529, Taiwan.

[b] Nano Science and Technology Program, Taiwan International Graduate Program, Academia Sinica and National Tsing Hua University

128, Section 2, Academia Road, Taipei 11529, Taiwan.

[c] Department of Engineering and System Science, National Tsing Hua University

101, Section 2, Kuang-Fu Road, Hsinchu 300044, Taiwan.

[d] Graduate Institute of Energy and Sustainability Technology, National Taiwan University of Science and Technology

43 Keelung Road, Sec 4, Taipei 10607, Taiwan

[e] Department of Applied Chemistry, National Chi Nan University,

1 University Road, Puli, Nantou, 545301, Taiwan

[f] College of Science, National Chung Hsing University

145 Xingda Rd., South Dist., Taichung City 402, Taiwan

*Corresponding author: **philwu@gmail.com** and **poweichi@gate.sinica.edu.tw**

Table of content

Figure S1. XRD patterns of AS200 with different single-layer coatings.

Figure S2. TEM images of AS200 with different single-layer coatings.

Figure S3. Charge-discharge curves of all samples at different C-rates within the voltage range of 2.2V to 4.6V.

Figure S4. Rate performance comparison of AS200 with 3nm-thick and 5nm-thick single-layer coatings. Charge-discharge curves at various C-rates provide insight into the electrochemical behavior.

Figure S5. Cycling performance of all samples under 1 C, along with corresponding charge-discharge curves at different cycles.

Figure S6. SEM image of all samples, capturing their morphological features both before and after 100 cycles at 0.1 C within the voltage range of 2.2V to 4.6V.

Figure S7. *Ex-situ* XRD patterns of all samples after 100 cycles at 0.1 C within the voltage range of 2.2V to 4.6V.

Figure S8. XPS spectrum of all samples following 100 cycles at 0.1 C within the voltage range of 2.2V to 4.6V.

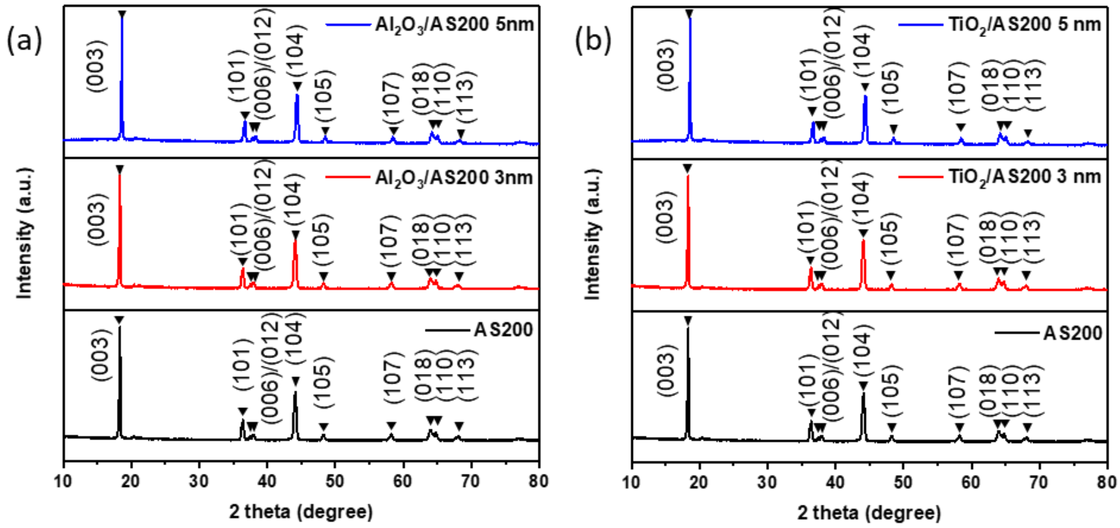

Figure S1. XRD patterns showing the structural characteristics of AS200 with different single-layer coatings. (a) Al_2O_3/AS200 and (b) TiO_2/AS200.

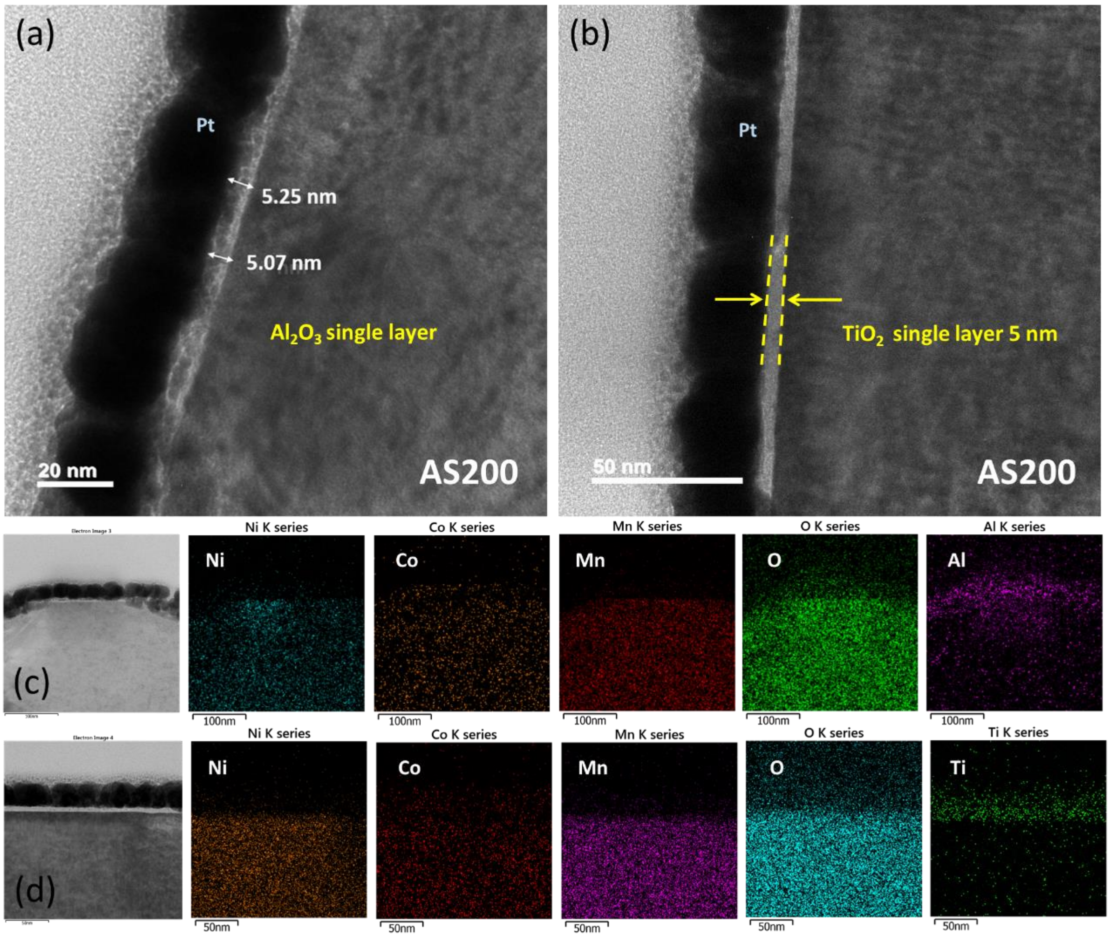

Figure S2. TEM images of AS200 with different single-layer coatings. (a) Al_2O_3/AS200 and (b) TiO_2/AS200. Corresponding elemental mapping images for (a) and (b) are presented in (c) and (d), respectively.

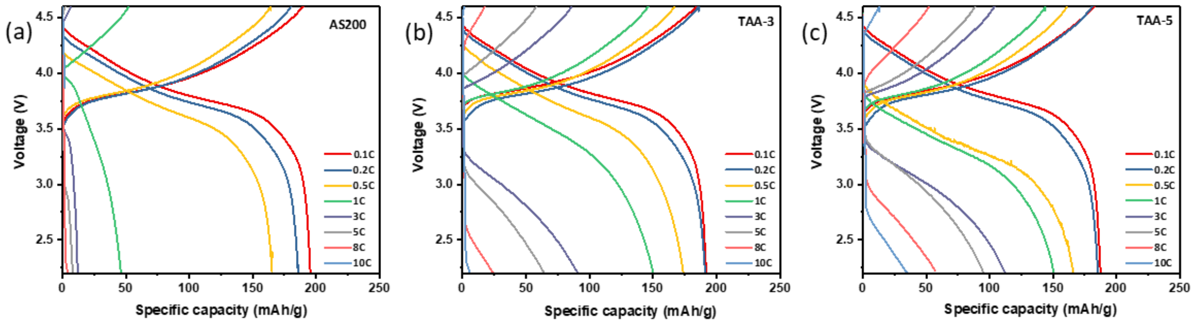

Figure S3. Charge-discharge curves (a) AS200, (b) TAA-3, and (c) TAA-5 under various C-rates. The potential range examined from 2.2V to 4.6V.

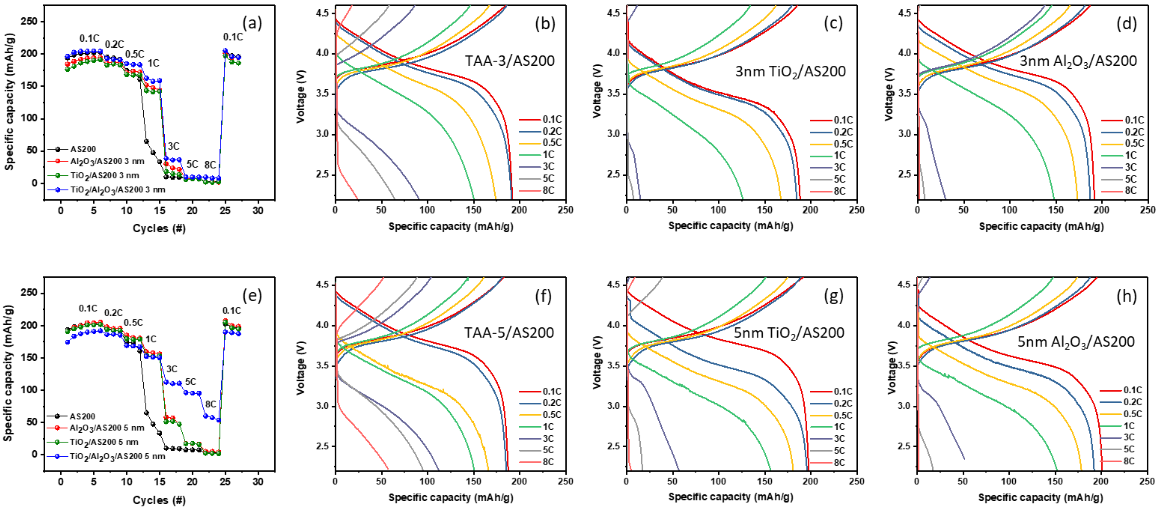

Figure S4. (a) Comparative rate performance of AS200, 3nm-TiO_2/AS200, 3nm-Al_2O_3/AS200, and TAA-3 electrodes under varying C-rates, with a potential range from 2.2V to 4.6V. Corresponding charge-discharge curves at different C-rates are presented for (b) TAA-3, (c) 3nm-TiO_2/AS200, and (d) 3nm-Al_2O_3/AS200. (e) Further comparative rate performance of AS200, 5nm-TiO_2/AS200, 5nm-Al_2O_3/AS200, and TAA-5 electrodes under varying C-rates, with a potential range from 2.2V to 4.6V. Corresponding charge-discharge curves at different C-rates are presented for (b) TAA-5, (c) 5nm-TiO_2/AS200, and (d) 5nm-Al_2O_3/AS200.

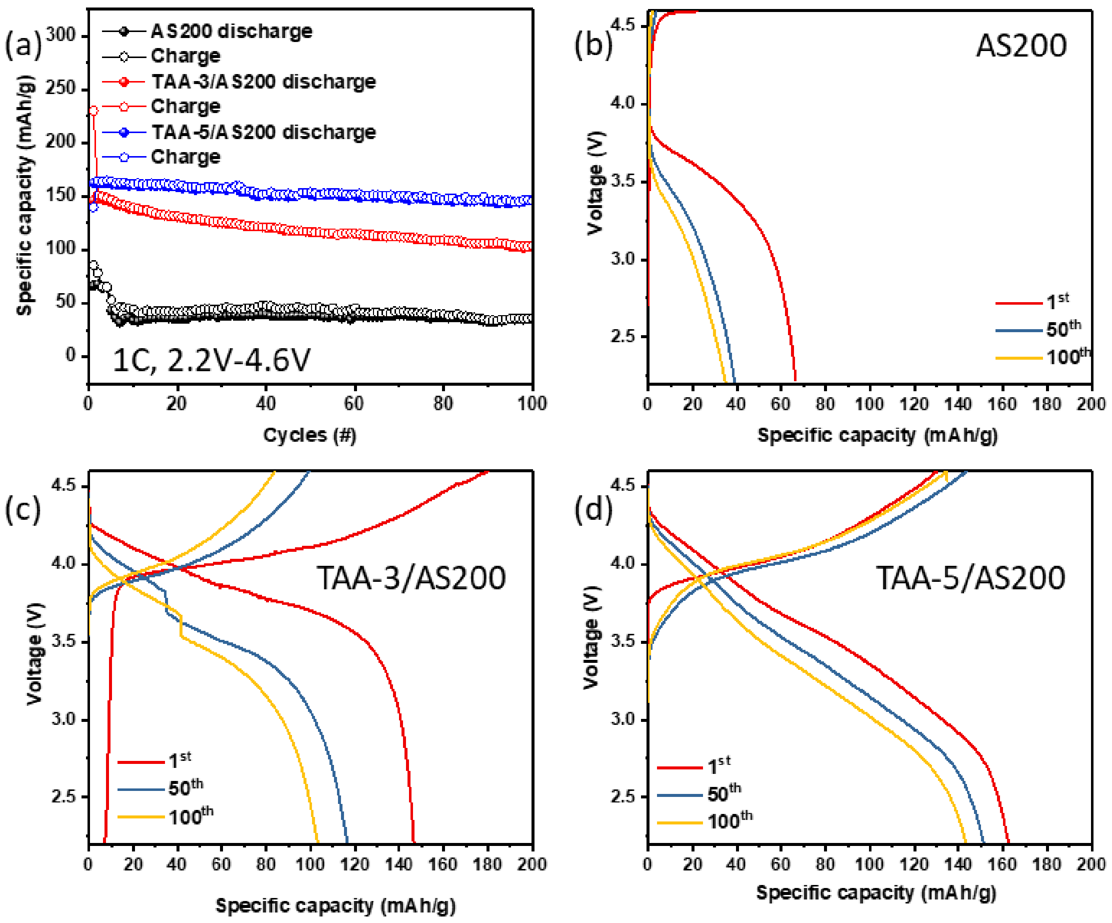

Figure S5. (a) Cycling performance comparison of AS200, TAA-3, and TAA-5 electrodes under 1 C, with a potential range spanning from 2.2V to 4.6V. The capacity retention after cycling for AS200, TAA-3, and TAA-5 electrodes is 52.5%, 70.6%, and 90.4%, respectively. Corresponding charge-discharge curves at different cycles are displayed for (b) AS200, (c) TAA-3, and (d) TAA-5 under 1 C.

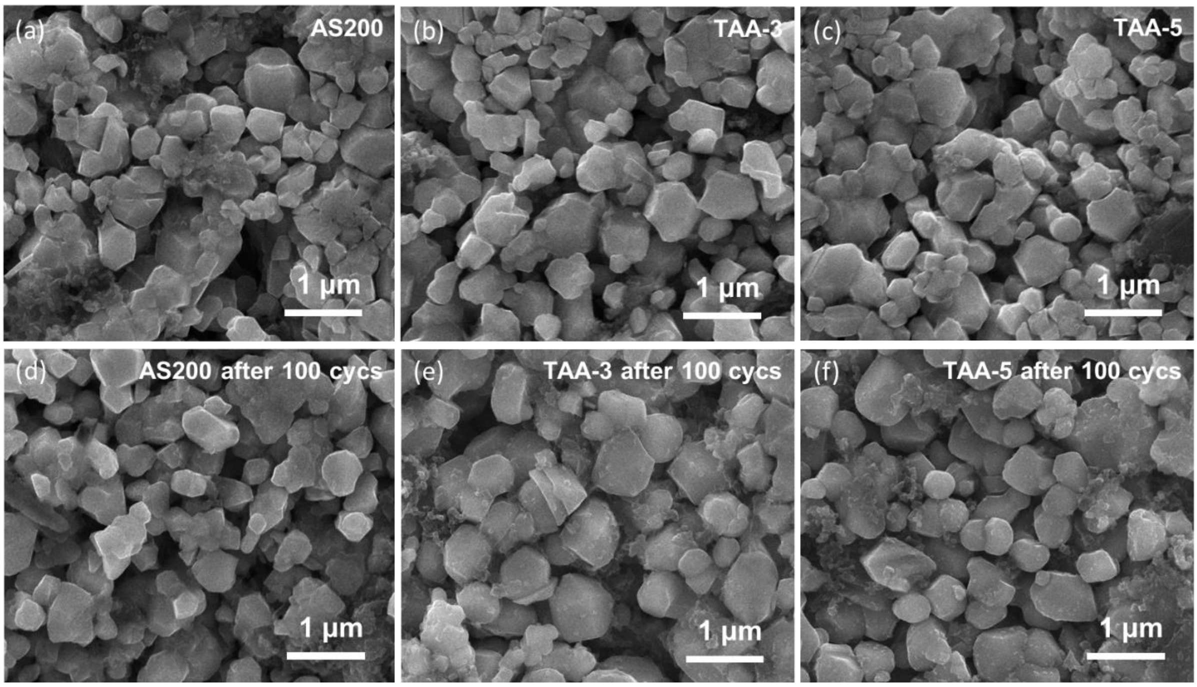

Figure S6. SEM image of (a) AS200, (b) TAA-3 and (c) TAA-5 before undergoing100 cycles. Subsequent SEM images, denoted as (d), (e), and (f), show the morphological changes after 100 cycles at 0.1 C within the voltage range of 2.2V to 4.6V.

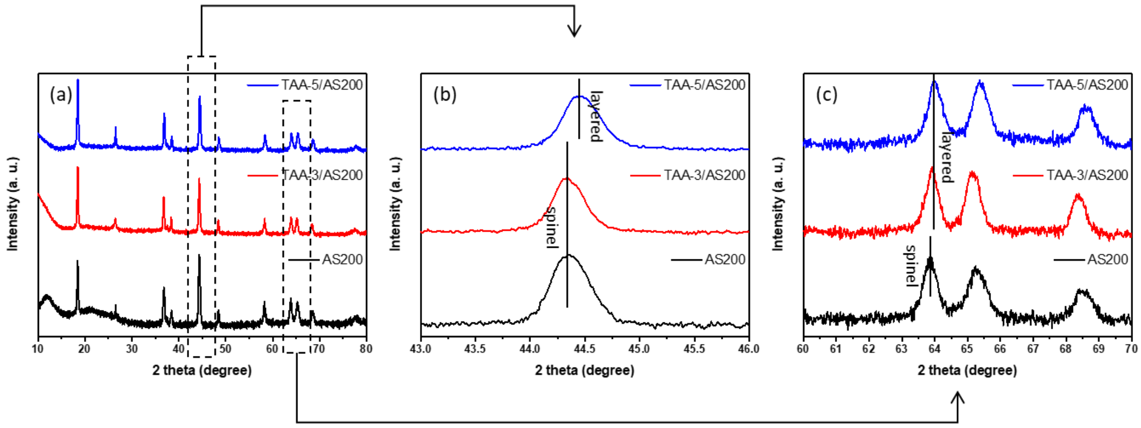

Figure S7. (a) *Ex-situ* XRD patterns of AS200, TAA-3, and TAA-5 electrodes after 100 cycles at 0.1 C within the voltage range of 2.2V to 4.6V. (b) provides a detailed magnification of XRD patterns between 43° to 46°, and (c) focuses on patterns within the range of 60° to 70°.

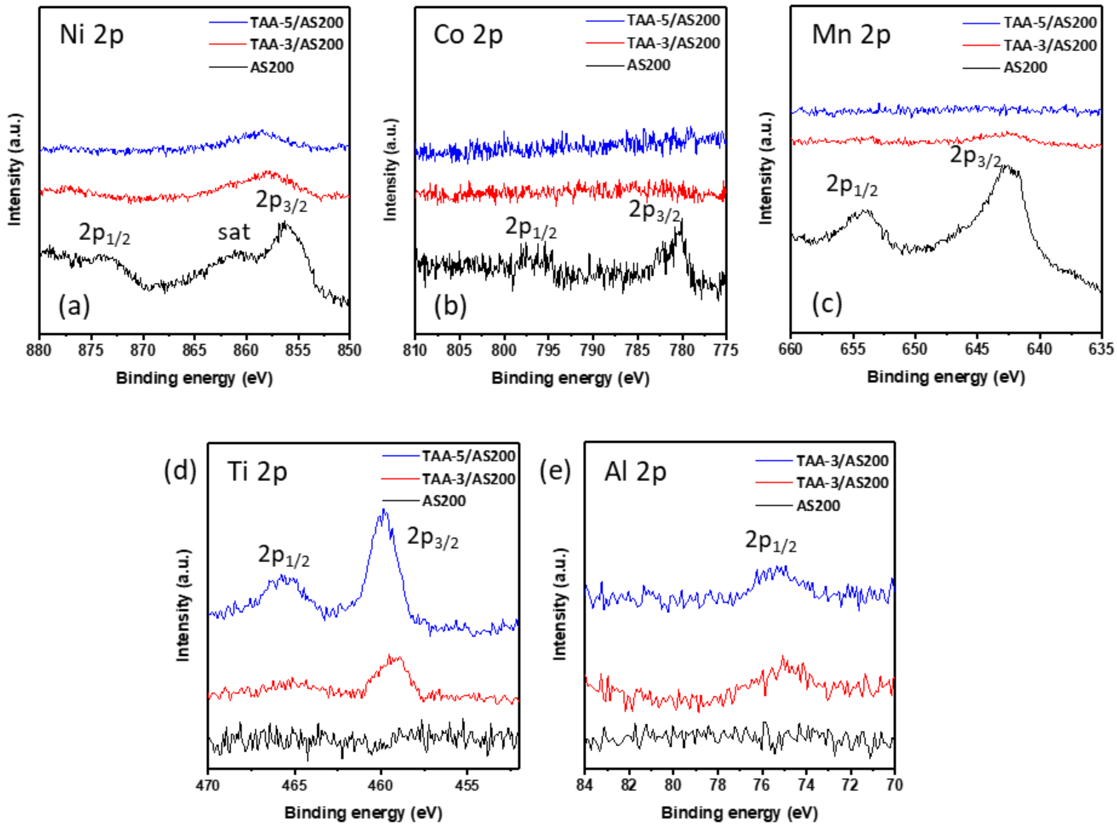

Figure S8. XPS spectrum of AS200, TAA-3, and TAA-5 electrodes following 100 cycles at 0.1 C within the voltage range of 2.2V to 4.6V. Individual spectra for (a) Ni 2p, (b) Co 2p, (c) Mn 2p, (d) Ti 2p, and (e) Al 2p are presented.
